# The prevalence of gestational syphilis in Malawi between 2014 and 2022: spatiotemporal modeling of population-level factors

**DOI:** 10.3389/fpubh.2023.1242870

**Published:** 2024-01-11

**Authors:** James Chirombo, Annielisa Majamanda, Vester Gunsaru, Simeon Yosefe, Washington Ozituosauka, Christina Mchoma, Chelsea Morroni, Effie Chipeta, Peter MacPherson, Bridget Freyne

**Affiliations:** ^1^Statistical Support Unit, Malawi Liverpool Wellcome Programme, Blantyre, Malawi; ^2^Department of Clinical Sciences, Liverpool School of Tropical Medicine, Liverpool, United Kingdom; ^3^Department of Quality, Blantyre District Health Office, Blantyre, Malawi; ^4^Digital Health Department, Ministry of Health, Lilongwe, Malawi; ^5^STI/HIV Program, Ministry of Health, Lilongwe, Malawi; ^6^Reproductive Health Department, Ministry of Health of Malawi, Lilongwe, Malawi; ^7^Botswana Harvard AIDS Institute Partnership, Gaborone, Botswana; ^8^MRC Centre for Reproductive Health, University of Edinburgh, Edinburgh, United Kingdom; ^9^Kamuzu University of Health Sciences, Blantyre, Malawi; ^10^School of Health and Wellbeing, University of Glasgow, Glasgow, United Kingdom; ^11^Clinical Research Department, London School of Hygiene and Tropical Medicine, London, United Kingdom; ^12^Department of Paediatric Infectious Diseases, Children’s Health Ireland, Dublin, Ireland; ^13^Division of Women and Children’s Health, School of Medicine, University College Dublin, Dublin, Ireland; ^14^Institute of Infection, Veterinary and Ecological Sciences, University of Liverpool, Liverpool, United Kingdom

**Keywords:** maternal syphilis, eMTCT, syphilis prevalence, spatio-temporal model, HIV

## Abstract

**Background:**

Mother-to-child transmission of syphilis remains high especially in the WHO AFRO region with a prevalence of 1.62%, resulting in a congenital syphilis rate of 1,119 per 100,000 live births. Elimination efforts can be supported by an understanding of the spatial and temporal changes in disease over time, which can identify priority areas for targeted interventions aimed at reducing transmission.

**Methods:**

We collated routine surveillance data from health facilities and covariate data from demographic and health surveys conducted in Malawi between 2014 and 2022. We fitted a Bayesian hierarchical mixed model with spatial and temporally structured random effects to model the district-level monthly counts of maternal syphilis notifications as a function of individual- and district-level predictors. We then generated district-level spatiotemporally explicit risk profiles to estimate the effect of individual- and district-level covariates on maternal syphilis notifications and to identify hotspot areas.

**Results:**

Overall, the national prevalence of maternal syphilis increased from 0.28% (95% CI: 0.27–0.29%) in 2014 to peaking in 2021 at 1.92% (95% CI: 1.89–1.96%). Between 2020 and 2022, there was a decline in prevalence, with the most significant decline seen in Zomba District (1.40, 95% CI: 1.12–1.66%). In regression models, a one percentage point increase in district-level antenatal HIV prevalence was associated with increased maternal syphilis (prevalence ratio [PR]: 1.15, 95% credible interval [CrI]: 1.10–1.21). There was also an increased prevalence of maternal syphilis associated with an increased district-level mean number of sex partners (PR: 1.05, 95% CrI: 0.80–1.37). The number of districts with a high prevalence of maternal syphilis also increased between 2014 and 2022, especially in the southern region, where most had a high probability (approaching 100%) of having high maternal syphilis (defined as relative risk >1 compared to the standard population of women aged 15–49 years) in 2022.

**Conclusion:**

Maternal syphilis prevalence in Malawi shows an increasing upward trend, with an estimated six times relative increase between 2014 and 2022 (0.28% to 1.73%) and strong associations with higher district-level HIV prevalence. Controlling syphilis depends on reaching vulnerable populations at the sub-national level, which may be disproportionately affected. Our findings support the move to integrate the elimination of mother-to-child transmission (EMTCT) of syphilis programs with existing prevention of mother-to-child transmission (PMTCT) of HIV programs.

## 1 Introduction

The World Health Organization (WHO) launched a campaign for the elimination of mother-to-child transmission (EMTCT) of syphilis in 2007 (1). EMTCT of syphilis has been defined as an incidence of <50 per 100,000 cases of congenital syphilis (CS). Although gains have been made over the last decade, the estimated global prevalence of maternal syphilis in 2016 was 0.69% (95% confidence interval [CI]: 0.51–0.87) leading to a global CS rate of 473 (385–561) per 100,000 live births and an estimated 661,000 (538,000-784,000) infants born with congenital syphilis per year, including 355,000 (290,000-419,000) adverse birth outcomes and a further 306,000 (249,000-363,000) asymptomatic infants at risk of neurodevelopmental and physical sequelae (2). These global estimates are almost double in the WHO AFRO region, where the maternal syphilis prevalence is estimated as 1.62% and CS rates at 1,119 per 100,000 live births (2).

In recent years, there has been a marked increase in notifications of early infectious maternal syphilis in high-income countries (3–5). High maternal syphilis prevalence is no longer limited to low-income countries. Congenital syphilis is also a re-emerging infection of global importance (6, 7). For example, countries, such as Japan, Australia, and New Zealand, have reported congenital syphilis outbreaks in recent years (8). In the USA, there has been a rise in congenital syphilis in both prevalence and geographical spread (6, 9).

Congenital syphilis is a devastating but entirely preventable disease. The risk of vertical transmission is significantly high in early infectious syphilis in the mother but can occur in secondary and latent syphilis. Untreated gestational syphilis will result in an adverse birth outcome in approximately 80% of cases (10). To achieve WHO accreditation for EMTCT of syphilis, a country must attain rates of 95% for each of (i) antenatal attendance, (ii) antenatal syphilis testing, and (iii) adequate treatment of pregnant women with benzathine penicillin >28 days pre-delivery (11). WHO guidelines for the prevention of mother-to-child transmission (PMTCT) of syphilis also include re-testing pregnant women in the third trimester, partner notification, and surveillance of congenital syphilis rates (12). A secondary analysis of Demographic and Health Survey (DHS) and Service Provision Assessment Survey (SPA) data recently indicated that only 23.8% of Malawian women were likely to access services in line with this recommendation (13). While major gains have been made in terms of the coverage of antenatal testing through the use of rapid point of care tests, there remain health systems and socio-cultural barriers to women accessing appropriate and timely treatment.

The WHO strategy for EMTCT of syphilis acknowledges that, in high prevalence settings, the elimination target of <50 per 100,000 cases of congenital syphilis/live births will be unattainable even with 95% coverage of testing and treatment (14). Treatment failure due to HIV co-infection, re-infection in late pregnancy, and treatment administered late in pregnancy will contribute to persistent congenital infection. The estimated antenatal prevalence of syphilis in Malawi is approximately 2% but has been reported as high as 8% in specific populations (15). Ultimately, combined approaches to interrupt community transmission of the disease alongside efforts to improve the management of infection in pregnancy will be required. Sub-national identification of areas of high prevalence of gestational syphilis may help to target resources aimed at the reduction of sexually transmitted infections in the community as well as areas of need for more intensive strategies for PMTCT.

Established risk factors for gestational syphilis infection in Africa include co-infection with HIV, living in urban centers, maternal education, maternal age, and key populations, including sex workers and lower socio-economic profile (16–18). In this analysis, we modeled the geographical variation in the risk of gestational syphilis over time. The aim was to identify sub-national areas of high syphilis risk and the relative contribution of currently measured and potentially modifiable risk factors.

## 2 Methods

### 2.1 Study design and setting

We designed a retrospective record review study using nationwide routine data collected by the Malawi Ministry of Health and stored in the District Health Information System (DHIS 2) database. Malawi is divided into 3 administrative regions, namely north, central, and southern regions, and further into 28 districts (see Supplementary Figure S1 for names and locations of all districts). Primary health care is provided by health posts, dispensaries, health centers, and rural/community hospitals, while district hospitals are secondary-level facilities that offer referral services for the district. Syphilis data are collected from the antenatal care clinics and later aggregated at the district level. Key district-level characteristics are shown in Supplementary Table S1.

### 2.2 Syphilis data

We obtained population-confirmed syphilis notification data (determined by the results of antenatal point-of-care testing) as counts per calendar month from the Malawi Ministry of Health’s Health Management Information System (HMIS). The notification data covered 108 months from 2014 to 2022 for all 28 Districts in Malawi. Screening using antibody tests is routinely done in pregnant women as it is in the Antenatal Care (ANC) package. Treponemal tests are the main tests used in Malawi. Venereal Disease Research Laboratory (VDRL) and rapid plasma reagin (RPR) tests are also used. Diagnosing congenital syphilis depends not only on the mother’s status but also on the clinical manifestations of the newborn baby, and it is treated asymptomatically by giving the infant 10 days of benzylpenicillin. The Malawi Standard Treatment Guidelines (MSTG) are followed in treating syphilis (19).

All confirmed syphilis-positive cases, regardless of the diagnostic method, were used in the analysis. The reporting rate in HMIS has steadily increased over the years. The rate is now at 90% from well-established government health facilities and private health facilities owned and operated by religious organizations with well-defined catchment areas (20). To estimate antenatal syphilis prevalence, we divided the number of positive maternal syphilis cases by the number of new ANC registrants. The syphilis test is done during the first antenatal visit, and in subsequent ANC visits, women are not recorded as new registrants.

### 2.3 District-level covariate data

Covariate data were obtained from the 2016 Demographic and Health Survey (DHS) and are outlined in Supplementary Table S2. Data including education, HIV status, median age at first delivery, number of sexual partners, and employment were accessed from the DHS program and then linked with the HMIS data by district. Because DHS estimates cover the preceding 5 years, we assumed that DHS estimates were constant during this study period. DHS surveys are large nationally representative surveys with large sample sizes conducted approximately every 5 years. DHS uses a two-stage sampling cluster survey strategy to select households. In the 2016 DHS, 26,361 households were included with 24,562 female respondents aged 15–49 years (21). The survey covered all 28 districts of Malawi. Data collected from households include employment, literacy, education, and household access to electricity among others. HIV testing was also done using enzyme-linked immunoassay (ELISA I). Samples testing positive on the ELISA I were subjected to ELISA II. Additional covariates from HMIS data included syphilis testing coverage, defined as the proportion of all women attending ANC that were tested. This syphilis rate was calculated by dividing the total number of syphilis tests done by the total number of ANC registrants.

### 2.4 Standardized incidence ratio

We calculated the standardized incidence ratio (SIR) to estimate the unadjusted risk of maternal syphilis in each district s at time t , which is given by


$$SIR_{st}=\frac{Y_{st}}{E_{st}}$$

Here, $Y_{st}$ is the observed number of maternal syphilis cases and $E_{st}$ is the expected number of cases defined as $\;E_{st}=r_{st}n_{st},$ where $r_{st}$ is the rate of maternal syphilis in the standard population and $n_{st}$ is the number of women of childbearing age in district s and month t . The maternal syphilis rate is $r_{st}=\frac{\sum Y_{st}}{\sum n_{st}}$ , calculated by dividing the total number of maternal syphilis cases by the total population of childbearing age. The ${\mathrm{SIR}}_{\mathrm{st}}$ approximates the district-specific relative risk in district s at time t . When ${\mathrm{SIR}}_{\mathrm{st}}>1$ , there is an elevated risk of maternal syphilis compared to the standard population of women of childbearing age, while ${\mathrm{SIR}}_{\mathrm{st}}<1$ denotes lower risk of maternal syphilis compared to the standard population. For calculating SIR, we standardized on female age only, thus giving unsmoothed estimate of risk. The spatiotemporal model, which was later fitted, allowed for the inclusion of covariates and spatial smoothing.

### 2.5 Model formulation

We assumed that the monthly maternal syphilis cases follow a Poisson distribution. To investigate factors affecting maternal syphilis risk, we, therefore, fitted a Bayesian hierarchical Poisson log-linear model with the following form:


$$\log\left(\mu_{st}\right)=\log\left(E_{st}\right)+\alpha +x_{st}^{T}\beta +\phi_{s}+\delta_{t}+\gamma_{st}$$

where $\mu_{st}$ is the mean number of maternal syphilis cases in district s and month t ; $E_{st}$ is the expected number of maternal syphilis cases and captures the possible differences in the characteristics of the women at the population level due to differences in the underlying population of women of childbearing age. The term α is the overall risk, and $x_{st}$ is the vector of p risk factors with their associated regression coefficient β. Finally, the terms $\phi_{s}$ , $\delta_{t},$ and $\gamma_{st}$ are the spatially structured, temporally structured, and the residual spatiotemporal random effects that capture extra Poisson variation or spatiotemporal correlation due to unmeasured risk factors. More details on the model specification are given in the Supplementary Material.

### 2.6 Model fitting

We first fitted non-spatial models with different combinations of covariates to explain maternal syphilis risk. We included the variable of testing coverage (defined as the number of all women attending ANC who were tested for syphilis divided by the total ANC registrants) to account for the increased testing rates over time. Model comparison was done using the Akaike information criterion (AIC). Overdispersion tests carried out on the preliminary models indicated the presence of overdispersion (extra-Poisson variation). The most parsimonious non-spatial GLM was then developed further by introducing both spatial and temporal random effects to account for the confirmed dispersion, as well as the possible presence of unobserved confounding factors. The spatially- and temporally structured random effects account for possible correlated random effects.

Parameter estimation was done in a Bayesian framework via Markov Chain Monte Carlo (MCMC). We fitted three parallel chains each with 620,000 iterations with a burn-in of 20,000 and a thinning parameter of 100, resulting in 6000 approximately independent samples from the joint posterior distribution for inference. Convergence was assessed visually by inspecting trace plots and analytically by computing the Geweke convergence diagnostic (22). All analyses were conducted in the R statistical environment for statistical computing (23).

### 2.7 Ethical considerations

We obtained approval from the Ministry of Health to access the DHIS 2 system and use the syphilis data for analysis. All the data in the DHIS 2 were aggregated with no individual identifying information. The DHS surveys, which provided the covariate data, already received ethical approval before they were conducted in Malawi.

## 3 Results

**Figure 1:**
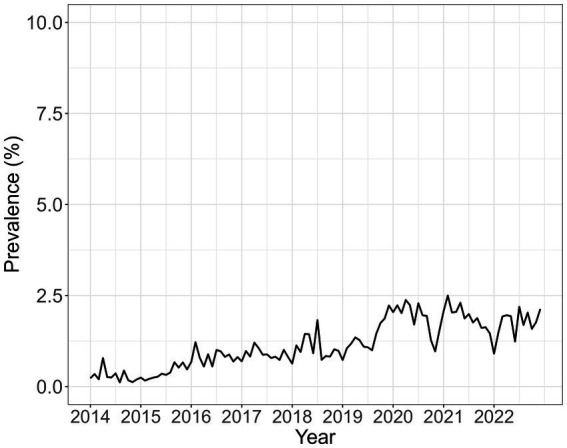
Empirical average monthly maternal syphilis prevalence among pregnant women attending ANC who completed testing for syphilis and were recorded in HMIS over the entire study period from 2014 to 2022. The prevalence was calculated by dividing the number of positive syphilis cases with the population of women registered for ANC.

Figure 1 shows the time series distribution of monthly maternal syphilis prevalence throughout the study period. Nationally, there has been an increase in prevalence among women tested from 0.23% (116 of 50,544) in January 2014 to 2.5% (1,220 of 48,795) in February 2021. From 2019, there have been episodes of marked alternating increase and decrease in prevalence.

**Figure 2:**
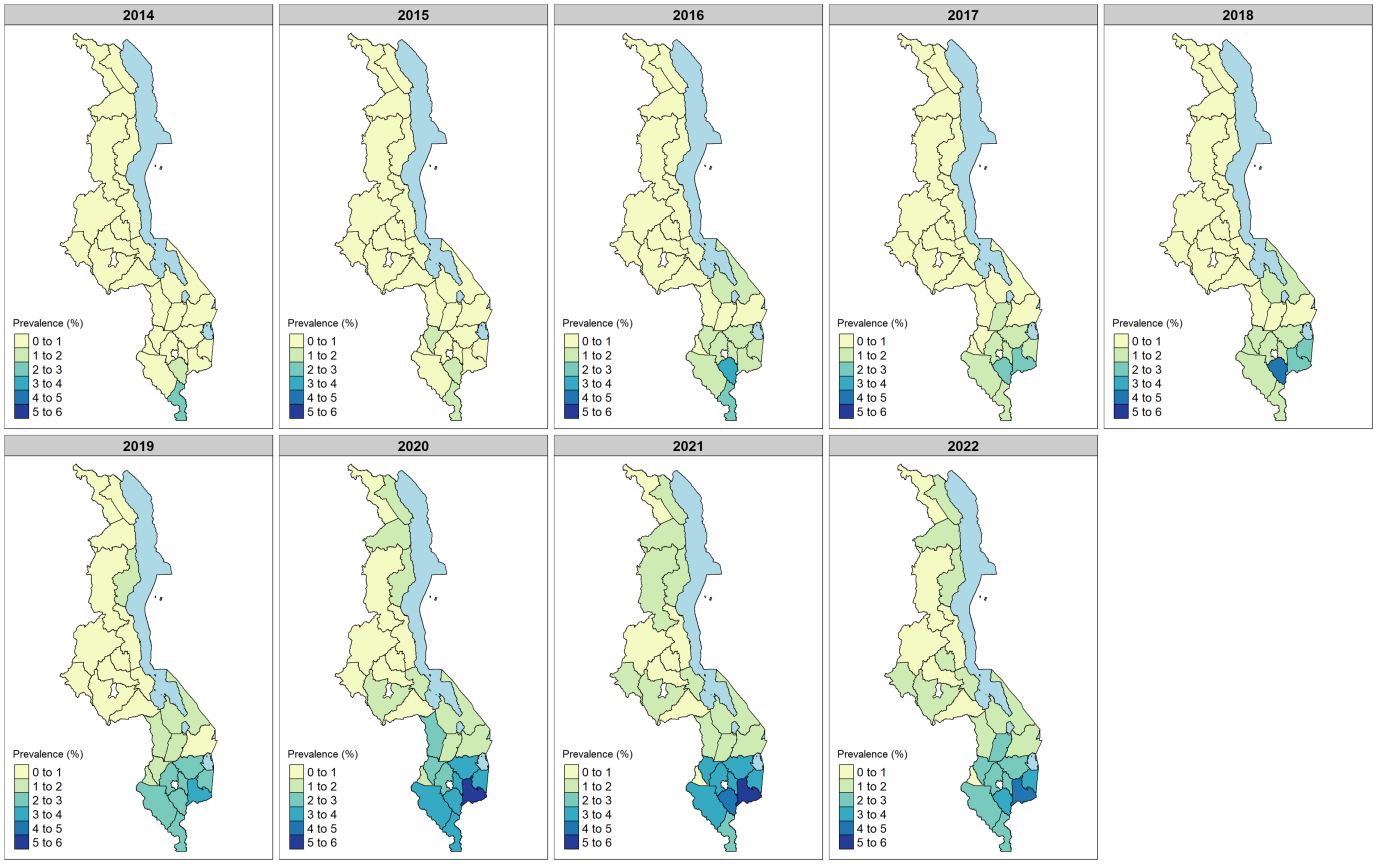
District-level summary of maternal syphilis empirical prevalence for the period of 2014–2022. The prevalence was calculated by dividing the number of positive syphilis cases with the population of women registered for ANC.

The changes in the spatial distribution of prevalence over time are shown in Figure 2. In general, prevalence has mainly increased mostly in the southern region. In 2014, there was a small number of districts with elevated prevalence, mostly in the southern region. By 2021/2022, most districts in southern Malawi had a prevalence ranging between 2% and 4%.

**Figure 3:**
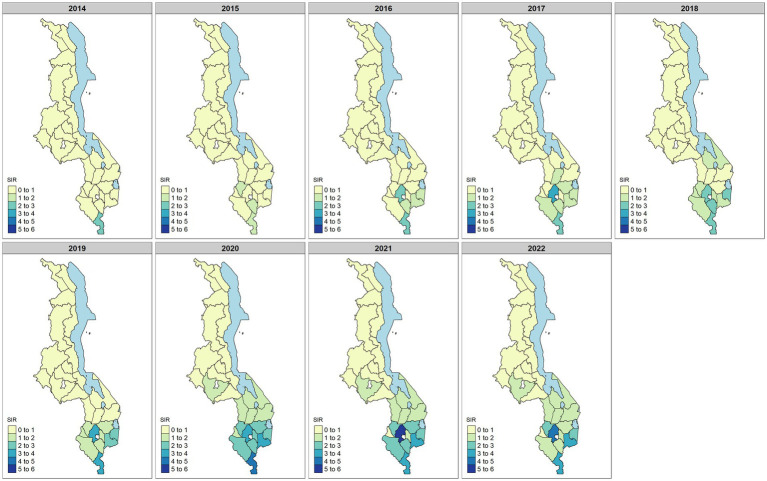
Standardized incidence ratio (SIR) estimates for maternal syphilis for the period of 2014–2022. Values SIR >1 indicate increased risk, while SIR <1 indicates decreasing risk of maternal syphilis.

Over time, the risk of syphilis, as estimated by the SIR, increased (Figure 3). The highest risk was generally found in several districts in the southern region. However, by 2022, there was a decrease in maternal syphilis prevalence in the southern region.

### 3.1 Factors affecting syphilis prevalence

Table 1 shows the model parameter estimates.

**Table 1:** Adjusted prevalence ratio estimates and their 95% credible intervals from the Poisson spatiotemporal model fitted to the data.

	Prevalence ratio	Lower bound of 95% CrI	Upper bound of 95% CrI
District % women employed	1.01	0.99	1.02
District % secondary education	0.87	0.80	0.98
District % testing coverage	1.02	0.80	1.02
District % electricity coverage	1.06	1.01	1.11
District median age birth	0.82	0.57	1.20
District % > 1 sex partner	1.05	0.80	1.37
District HIV prevalence	1.15	1.10	1.21

* Adjusted prevalence ratio estimates obtained from exponentiating the coefficients of the spatiotemporal model.

**Figure 4:**
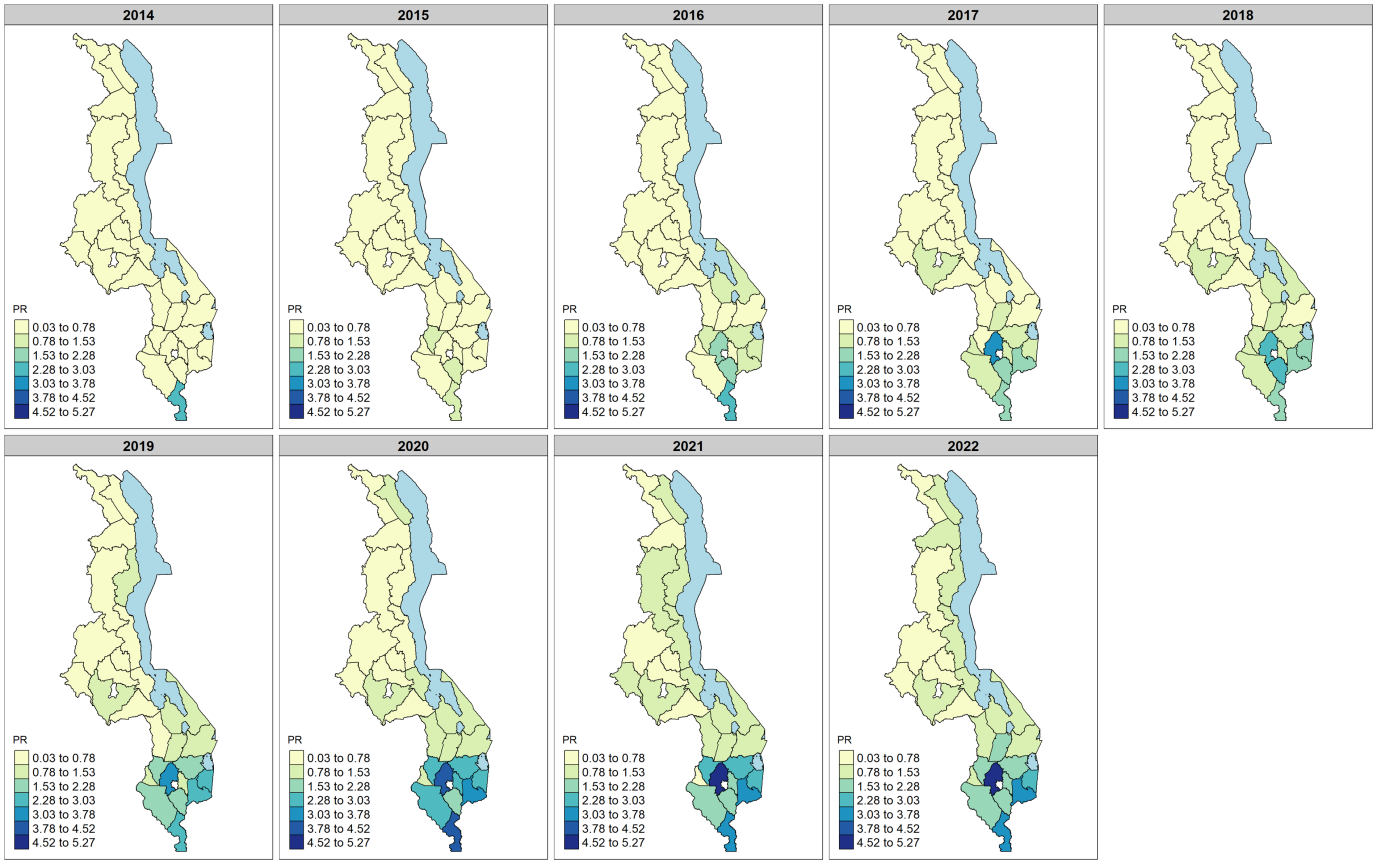
Predicted district maternal syphilis prevalence ratio (PR) over the period of 2014–2022. The predicted values are obtained from the fitted model. Darker colors on the map indicate districts with a high predicted prevalence ratio, while lighter colors indicate districts with a lower predicted prevalence ratio.

From the model, a one percentage point increase in the percentage of women reporting being employed in the last 12 months did not lead to a change in maternal syphilis (prevalence ratio [PR]: 1.01, 95% CrI: 0.99–1.02). However, a one percentage point increase in the district-level proportion of households with access to electricity was associated with a 6% relative increase in the risk of maternal syphilis in pregnancy (PR: 1.06, 95% CrI: 1.01–1.11). Furthermore, for a one percentage point increase in 15–49-year-old women who reported having completed secondary-level education, there was a 13% (PR: 0.87, 95% CrI: 0.80–0.98) decrease in the risk of maternal syphilis. A one percentage point increase in the district-level percentage of women reporting more than one sexual partner was not associated with an increased risk (PR: 1.05, 95% CrI: 0.80–1.37); each percentage point in district-level HIV prevalence among women aged 15–49 years was associated with a 15% (PR: 1.15, CrI: 1.10–1.21) increase in the risk of maternal syphilis. The annual predicted district syphilis risk based on the model is shown in Figure 4.

### 3.2 Model-predicted syphilis risk

**Figure 5:**
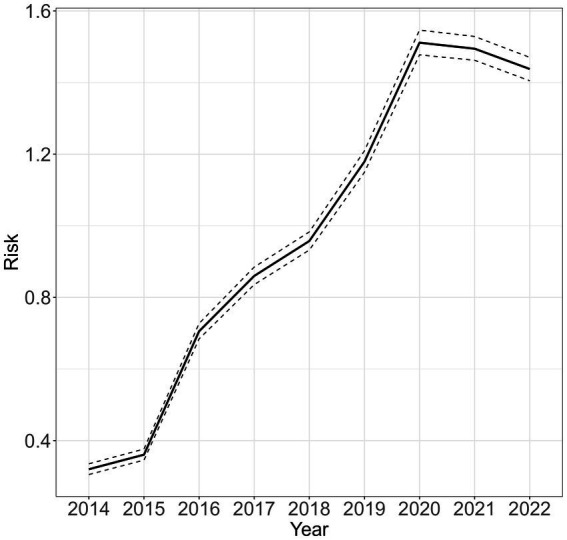
Average yearly maternal syphilis risk for the entire country between 2014 and 2022 relative to the standard population of women aged 15–49 years in the corresponding year. Values greater than 1 indicate a higher risk compared to the standard population.

The model predicted a higher risk of maternal syphilis in most of the districts in the southern region, and there was an observed increase in the number of districts with a high prevalence of maternal syphilis. For example, Nsanje was the only district in the south with a PR of >1.5 in 2014, but by 2022, 10 districts in the southern region (Balaka, Zomba, Mulanje, Phalombe, Mwanza, Chikwawa, Nsanje, Blantyre, Thyolo, and Chiradzulu) had PR > 1.5 (see Figure 4). The temporal changes in the risk also indicate a slow rate of increasing risk of maternal syphilis from 0.2 in 2014 to 1.5 in 2020 (Figure 5). From 2020 to 2022, there was a slight decrease in the risk, from 1.5 to 1.44.

**Figure 6:**
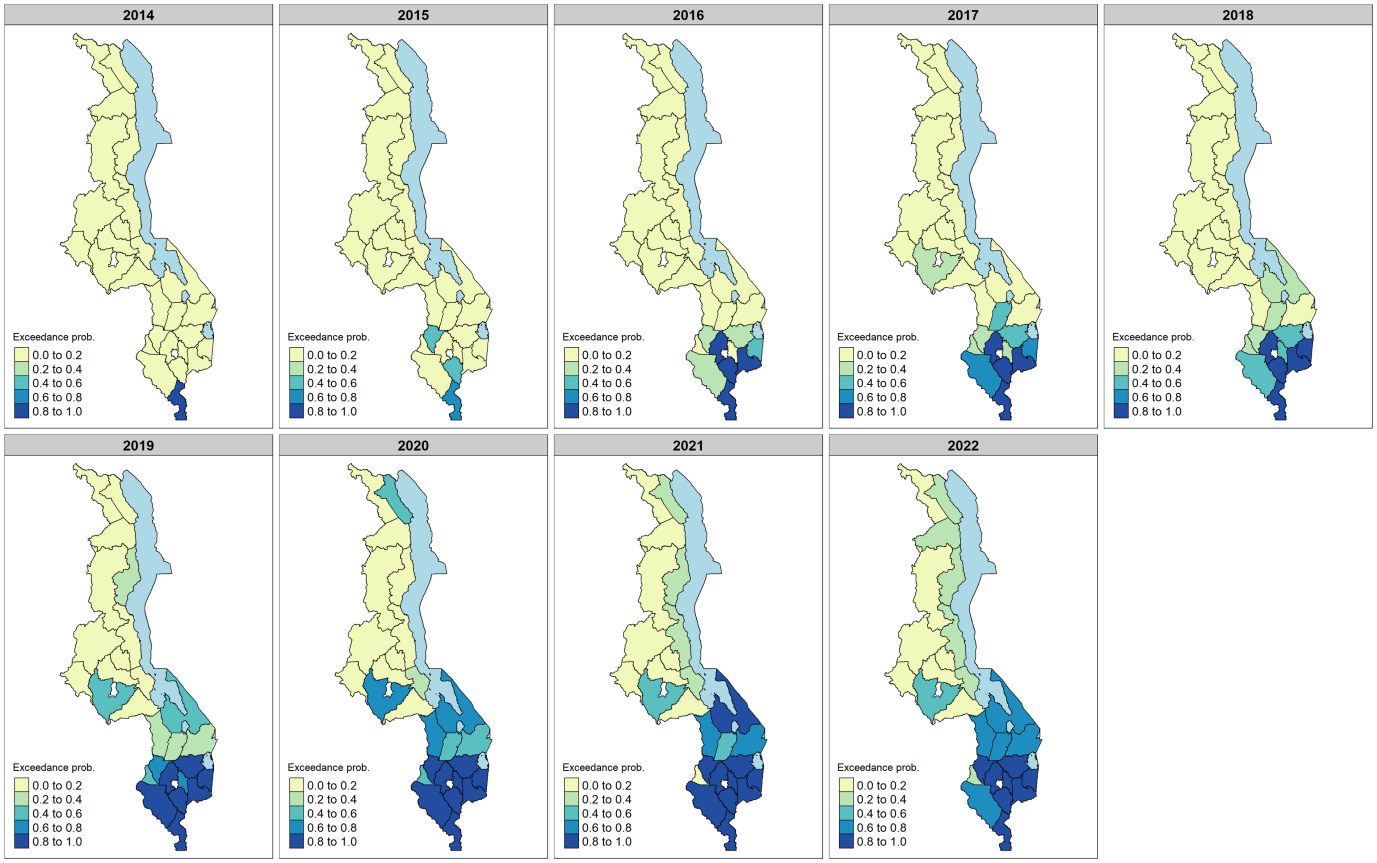
Posterior exceedance probabilities that the risk is greater than 1 between 2014 and 2022. The probabilities were calculated from the model. Darker colors on the map indicate higher probabilities with values close to 1, especially in the southern region.

Finally, we calculated the posterior exceedance probabilities $P\left(\theta_{i}>c\right)$ that the prevalence ratio (PR) in district s is greater than a pre-specified value c . We used a threshold value of 1 to find high-risk districts. Most of the districts in the Northern and Central regions had low probabilities of having a higher risk greater than one, with most districts having a probability less than 0.2 (Figure 6). In later years, the probability slightly increased mostly in districts along the lake, such as Nkhotakota and Salima. In the southern region, the number of districts with high probabilities of having increased maternal risk increased from one district in 2014 to 12 districts in 2022. The number of districts with the highest risk in the south was maximum in 2021 before slightly decreasing in 2022. Districts with the highest risk in this region include Thyolo, Mulanje, Nsanje, and Phalombe.

## 4 Discussion

To the best of our knowledge, this is the first report of the geospatial distribution of maternal syphilis in an African country using routinely collected health system data. This method has been extensively used to map the evolving problem of syphilis in pregnant women and their infants in both the United States (24, 25) and Brazil (26–33). Leveraging routinely collected data in this way provides a return for investment on district-level data collection. It further supports the development of “precision” public health, which seeks to harness epidemiological data for optimal allocation of available resources.

High-quality district-level data is required to support the development of a national strategy with the aim of attaining international health policy targets. In Brazil, regional-level data identified regions with high migration and high tourist levels as “hotspots” for congenital syphilis (34). In China and Brazil, geospatial methods identified divergence in the development of syphilis and HIV epidemics (35, 36), while in South Africa, a cross-sectional spatial analysis of HIV and syphilis hotspots also showed that they did not overlap (37). In China, syphilis prevalence was associated with areas of rapid economic development (38). In our analysis, we identified a trend of increasing syphilis prevalence over time, which was concentrated in the southern region of Malawi and along the lake shore. It is possible that this is due to the higher levels of urbanization, the presence of major trade routes, and increased migration for work and trade in these areas, but additional targeted surveillance would be required to further explore these possibilities.

By utilizing available district-level demographic data, it is possible to explore risk factors within a population that may contribute to a relatively higher risk of maternal syphilis in that region. In Malawi, regions with a higher HIV prevalence among women of childbearing age also exhibited a higher risk of maternal syphilis. This is in keeping with a 2010 geospatial analysis of HIV prevalence in Malawi, which showed a similar increased risk of HIV in the southern region (39). Based on these observations, the proposed integration of EMTCT programs for HIV and syphilis should be effective in Malawi (40). Our data also support the notion that fewer years of maternal education was associated with an increased risk of maternal syphilis in Malawi. Similar associations have been noted with epidemiological analyses from Brazil (31, 41, 42). Economic and cultural factors that contribute to vulnerability with respect to the sexual health of young women may be different in Malawi and visualizing this by region can support the need for targeted interventions in certain regions.

The observed plateau and decrease in syphilis risk between 2020 and 2022 could have been partly due to the COVID-19 pandemic, which caused some disruptions to the health service, leading to a decrease in syphilis notifications across the country. Decreases in syphilis notifications attributable to the pandemic have been observed in some settings (43, 44). However, more research works are needed to ascertain the true cause of the decrease in the risk in Malawi.

The major strength of this study is the wide geographical distribution of health facilities from which data is drawn, which provides confidence in the representativeness of our results. A further strength is the modeling approach used, which takes into consideration the spatial and temporal structure of the data so that the estimates are more likely to capture underlying heterogeneities between regions. The major limitation of this study relates to the quality of the data used. Since routine HMIS data are dependent on health facilities reporting their data into the central system, missing data are not uncommon and mostly affect small rural health facilities that may not have adequate infrastructure and personnel. We have mitigated this problem as much as possible by doing a district-level analysis using a Bayesian analytical approach, but the results of the study need to be interpreted in light of this limitation. Another limitation is the use of DHS estimates, which assumed that covariate values were constant over the study period. A further limitation of the HMIS data is the inability to capture certain syphilis-related outcomes. For example, gestational syphilis case data may not be representative as pregnant women not presenting at the facility for ANC visits are not included in our estimates. An important limitation of this data is that current standard antenatal testing for syphilis in Malawi is a Treponemal-only rapid Point-of-Care Testing (POCT), but we cannot rule out VDRL or RPR use in some centers in earlier years. We do not have clinical information to confirm the stage or acuity of infection. In addition, there is no existing surveillance for congenital syphilis at the time of delivery in the current dataset analyzing this outcome of EMTCT impossible.

## 5 Conclusion

The results of this study highlight the importance of high-quality district-level data to support precision public health that can be improved by employing low-cost regular and consistent auditing of the routine data as a way of improving the quality and investing in electronic data capture at the point of care to minimize data entry errors. Presently, data entry at source is still paper based. This approach may be particularly valuable in low- and middle-income countries where resources are scarce, and interventions need to be targeted for maximum effect. The results of this study need to be interpreted considering the limitations discussed and should be used to support the design of definitive epidemiological studies. Based on our results, future areas of study should target vulnerable young women, be integrated with HIV services, and be focused on regions of rapid development and high migration in Malawi. Modification to the HMIS dataset to enable surveillance of congenital syphilis that links STI and antenatal clinic data is urgently required, as is an investment into data quality and completeness at the district level.

## Data availability statement

The original contributions presented in the study are included in the article/Supplementary material, further inquiries can be directed to the corresponding author.

## Author contributions

BF conceived the study. JC and PM also helped in conceiving the study. JC collated the data, analyzed the results, and fitted the models. VG and PM provided advice on statistical modeling and critically reviewed the methods. AM, SY, WO, CMc, CMo, and EC provided insight into maternal syphilis in Malawi. SY authorized the use of DHIS data and clarified data collection systems. All authors contributed to the article and approved the submitted version.
