# Supplementary material for: The prevalence of gestational syphilis in Malawi between 2014 and 2022: spatiotemporal modeling of population-level factors
Source: Front Public Health. 2024 Jan 11;11:1242870. doi: 10.3389/fpubh.2023.1242870 (PMC10825961; doi:10.3389/fpubh.2023.1242870)
Supplement: Supplementary file 1 [file Table_1.DOCX]

Supplementary Material

The prevalence of gestational syphilis in Malawi between 2014-2022: spatio-temporal modelling of population level factors

James Chirombo^1,2^, Annielisa Majamanda^3^, Vester Gunsaru^1^, Simeon Yosefe^4^, Washington Ozituosauka^5^, Mchoma C^6^, Morroni C^7,8^, Effie Chipeta^9^, Peter MacPherson^10,11^, Bridget Freyne^12,13*^

*** Correspondence:** Bridget Freyne: [bridget.freyne@ucd.ie](mailto:bridget.freyne@ucd.ie)

## Model formulation

To investigate factors affecting maternal syphilis risk, we used a Bayesian hierarchical Poisson log-linear model. Let $Y_{st}$ be the number of maternal syphilis cases in district $s=1,2,\ldots,27$ and month $t=1,2,\ldots,108$. We excluded Likoma, an island district in Lake Malawi to obtain a contiguous Malawi region thus yielding 27 districts for modelling. For rare conditions in the population, the Poisson distribution approximates the underlying binomial distribution for estimating the risk in each unit $s$ at time $t$. Then $Y_{st}$ follows a Poisson distribution as follows

|  | $\boldsymbol{Y}_{\boldsymbol{st}}\boldsymbol{\sim}\mathbf{Poisson}\boldsymbol{(}\boldsymbol{\mu}_{\boldsymbol{st}}\boldsymbol{=}\boldsymbol{E}_{\boldsymbol{st}}\boldsymbol{R}_{\boldsymbol{st}}$**)** | (1) |
| --- | --- | --- |

where the mean $\mu_{st}=E_{st}R_{st}$. The variable $E_{st}$ is the expected number of maternal syphilis cases in district $s$ and month $t$ as described in equation 1 and captures the possible differences in the characteristics of the women at the population level due to differences in the underlying population of women of childbearing age. The term, $R_{st}$ is the maternal syphilis risk for district $s$ at time $t$ on the same scale as the SIR. The log of the relative risk term, $\log R_{st}$ is then specified as a linear combination of District-level risk factors and both spatial and temporal random effects.

Following on from equation 2, the final model was defined as follows:

|  | $\log\left( \boldsymbol{\mu}_{\boldsymbol{st}} \right)\boldsymbol{=}\log\left( \boldsymbol{E}_{\boldsymbol{st}} \right)\boldsymbol{+}\log\left( \boldsymbol{R}_{\boldsymbol{st}} \right)$ | (2) |
| --- | --- | --- |

Presenting the log relative risk term as a linear combination of covariates and random effects:

|  | $\mathbf{log}\left( \boldsymbol{R}_{\boldsymbol{st}} \right)\boldsymbol{=\alpha+}\boldsymbol{x}_{\boldsymbol{st}}^{\boldsymbol{T}}\boldsymbol{\beta+}\boldsymbol{\phi}_{\boldsymbol{s}}\boldsymbol{+}\boldsymbol{\delta}_{\boldsymbol{t}}\boldsymbol{+}\boldsymbol{\gamma}_{\boldsymbol{st}}$ | (3) |
| --- | --- | --- |

Where $\alpha$ is the overall risk, $\boldsymbol{x}_{\boldsymbol{st}}\boldsymbol{=}\left( x_{st1},\ldots,x_{stp} \right)$ is the vector of $p$ risk factors with their associated regression coefficient $\boldsymbol{\beta=}\left( \beta_{1},\ldots,\beta_{p} \right)$. The terms $\phi_{s}$, $\delta_{t}$ and $\gamma_{st}$ are the spatially structured, temporally structured and the residual spatio-temporal random effects that capture extra Poisson variation or spatio-temporal correlation due to unmeasured risk factors. Our full model then takes the form:

|  | $\mathbf{log(} \boldsymbol{\mu}_{\boldsymbol{st}}\boldsymbol{)=}\log\left( \boldsymbol{E}_{\boldsymbol{st}} \right)\boldsymbol{+\alpha+}\boldsymbol{x}_{\boldsymbol{st}}^{\boldsymbol{T}}\boldsymbol{\beta+}\boldsymbol{\phi}_{\boldsymbol{s}}\boldsymbol{+}\boldsymbol{\delta}_{\boldsymbol{t}}\boldsymbol{+}\boldsymbol{\gamma}_{\boldsymbol{st}}$ | (4) |
| --- | --- | --- |

The spatial random effect, $\phi=\left( \phi_{1},\ldots,\phi_{s} \right),$and the temporal random effect $\delta=\left( \delta_{1},\ldots,\delta_{t} \right)$ form Gaussian Markov random fields (GMRF). The spatio-temporal effects are mutually independent with the distribution $\gamma_{st} \sim N\left( 0,\tau_{I}^{2} \right)$.

The spatially structured random effects were specified through the conditional autoregressive model (CAR) as proposed by Leroux (22). In this formulation, the random effects “borrow” the strength from neighbouring districts leading to the smoothing of the risk. This is because districts near each other are likely to be similar than districts further apart. We defined a symmetric $m\times m$ neighourhood matrix $\boldsymbol{W}$ with elements $w_{ij}=1$ if districts $i$ and$j$are neighbours and 0 otherwise. Any two districts $i$ and $j$ were neighbours if they shared a common boundary.

Similarly, the temporally-structured random effects were modelled using a CAR model to capture temporal effects, which are more similar for adjacent time points compared to time points that are further apart. The temporal relationships are captured by a symmetric $n\times n$ matrix $\boldsymbol{V}$ with elements $v_{ij}$. We specified $v_{ij}=1$ if months $i$ and $j$ are adjacent such that $\left| j-i \right|=1$ and $v_{ij}=0$ otherwise. The full conditional distributions for the spatial and temporal effects are:

|  | $\boldsymbol{\phi}_{\boldsymbol{s}}\boldsymbol{\vert}\boldsymbol{\phi}_{\boldsymbol{-s}}\boldsymbol{\sim N}\left( \frac{\boldsymbol{\rho}_{\boldsymbol{S}}\sum_{\boldsymbol{j=1}}^{\boldsymbol{m}} \boldsymbol{w}_{\boldsymbol{sj}}\boldsymbol{\phi}_{\boldsymbol{j}}}{\boldsymbol{\rho}_{\boldsymbol{S}}\sum_{\boldsymbol{j=1}}^{\boldsymbol{m}} \boldsymbol{w}_{\boldsymbol{sj}}\boldsymbol{+1-}\boldsymbol{\rho}_{\boldsymbol{S}}}\boldsymbol{,}\frac{\boldsymbol{\tau}_{\boldsymbol{S}}^{\boldsymbol{2}}}{\boldsymbol{\rho}_{\boldsymbol{S}}\sum_{\boldsymbol{j=1}}^{\boldsymbol{m}} \boldsymbol{w}_{\boldsymbol{sj}}\boldsymbol{+1-}\boldsymbol{\rho}_{\boldsymbol{S}}} \right)$ | (5) |
| --- | --- | --- |

and

| $\boldsymbol{\delta}_{\boldsymbol{t}}\boldsymbol{\vert}\boldsymbol{\delta}_{\boldsymbol{-t}}\boldsymbol{\sim N}\left( \frac{\boldsymbol{\rho}_{\boldsymbol{T}}\sum_{\boldsymbol{j=1}}^{\boldsymbol{n}} \boldsymbol{v}_{\boldsymbol{tj}}\boldsymbol{\delta}_{\boldsymbol{j}}}{\boldsymbol{\rho}_{\boldsymbol{T}}\sum_{\boldsymbol{j=1}}^{\boldsymbol{n}} \boldsymbol{v}_{\boldsymbol{tj}}\boldsymbol{+1-}\boldsymbol{\rho}_{\boldsymbol{T}}}\boldsymbol{,}\frac{\boldsymbol{\tau}_{\boldsymbol{T}}^{\boldsymbol{2}}}{\boldsymbol{\rho}_{\boldsymbol{T}}\sum_{\boldsymbol{j=1}}^{\boldsymbol{n}} \boldsymbol{v}_{\boldsymbol{tj}}\boldsymbol{+1-}\boldsymbol{\rho}_{\boldsymbol{T}}} \right)$ | **(6)** |
| --- | --- |

Both the spatial and temporal random effects were mean centred. We used weakly informative priors for the fixed effects $\beta\sim N\left( 0,1000 \right)$ . We then assigned independent inverse gamma priors $\tau^{2} \sim Inverse-Gamma\left( 1,0.001 \right)$ to the variance components $\tau_{S}^{2}, \tau_{T}^{2}$ and $\tau_{I}^{2}$. Lastly, we assigned independent uniform priors $\rho\sim U\left( 0,1 \right)$ to the autocorrelation parameters $\rho_{S}$ and $\rho_{T}$.

# Supplementary Figures and Tables

Supplementary Table S1: A summary of key district-level demographic characteristics of women aged 15-49 years for all districts.

|  | % employed | % with secondary education | % Syphilis testing coverage | % households with electricity | Median age at first birth | % more than 1 sex partner | % HIV+ | % with STI infection |
| --- | --- | --- | --- | --- | --- | --- | --- | --- |
| Balaka | 33.9 | 5.6 | 76.9 | 6.1 | 18.8 | 1.6 | 4.4 | 3.2 |
| Blantyre | 52 | 16.5 | 59 | 40.2 | 19.2 | 1.9 | 12.9 | 1.6 |
| Chikwawa | 45.7 | 3.5 | 59.2 | 5.4 | 18.9 | 1.1 | 6.5 | 5 |
| Chiradzulu | 27.1 | 3.6 | 66.7 | 4.2 | 18.7 | 0.3 | 4.7 | 2.6 |
| Chitipa | 28.2 | 6.7 | 52 | 7.9 | 18.8 | 1.3 | 2.5 | 1.6 |
| Dedza | 9 | 1.7 | 73.8 | 1.3 | 19.4 | 0.5 | 1.9 | 1.9 |
| Dowa | 31.8 | 4.7 | 60.6 | 4 | 19.9 | 0.5 | 2.7 | 4.9 |
| Karonga | 41.8 | 6.5 | 63.4 | 8.4 | 18.5 | 0.6 | 8.7 | 2.1 |
| Kasungu | 24.8 | 5.8 | 65 | 5.9 | 19.4 | 1.6 | 2 | 2.4 |
| Likoma | 52.1 | 11.6 | 62.5 | 44.7 | 19.4 | 0.8 | 5.1 | 0.1 |
| Lilongwe | 19.5 | 9.7 | 69.9 | 16.9 | 19.8 | 1.8 | 6.5 | 2.6 |
| Machinga | 27.3 | 2.1 | 72.3 | 4 | 18.5 | 1 | 3.1 | 1.3 |
| Mangochi | 58.9 | 2.5 | 68.6 | 7 | 18.5 | 1.8 | 5.7 | 2.3 |
| Mchinji | 9.5 | 3 | 75.2 | 3.3 | 19.1 | 1.9 | 4.8 | 2 |
| Mulanje | 29.3 | 2.7 | 74.1 | 7.9 | 18.3 | 0.8 | 14.2 | 4.1 |
| Mwanza | 42.1 | 6 | 59 | 13 | 19.1 | 2 | 3.2 | 2.9 |
| Mzimba | 51.2 | 7.4 | 73.3 | 12.8 | 19.1 | 1 | 3.1 | 3.2 |
| Neno | 37 | 4.8 | 82.9 | 3.7 | 18.6 | 0.6 | 9.8 | 3.9 |
| Nkhatabay | 29.2 | 6.3 | 75.3 | 12.5 | 19.1 | 1.5 | 6.1 | 2.3 |
| Nkhotakota | 46.2 | 8 | 68.1 | 9.4 | 19.6 | 1.1 | 7 | 2.5 |
| Nsanje | 37.1 | 2.6 | 57.1 | 7.4 | 19.1 | 2.2 | 9.5 | 3.1 |
| Ntcheu | 19.5 | 4.6 | 75.9 | 4.4 | 18.9 | 0.6 | 3.7 | 0.7 |
| Ntchisi | 52.8 | 5.3 | 62.3 | 6.7 | 19.7 | 1.4 | 3.4 | 2.5 |
| Phalombe | 15.2 | 2 | 67.3 | 2.7 | 18 | 1.8 | 11.6 | 4.9 |
| Rumphi | 29.4 | 9.1 | 73.5 | 14.9 | 18.9 | 2.4 | 5.5 | 2.8 |
| Salima | 35.3 | 3.6 | 65.6 | 6 | 18.8 | 0.7 | 1.8 | 1.7 |
| Thyolo | 33.5 | 5.5 | 75.3 | 6.9 | 18.6 | 0.3 | 11.2 | 2.9 |
| Zomba | 34.2 | 7.3 | 71.5 | 11.7 | 18.5 | 0.8 | 9.3 | 5 |

Supplementary Table S2: Variable description and their sources

| **Covariate** | **Description** | **Variable type** | **Source** |
| --- | --- | --- | --- |
| Antenatal syphilis cases | Positive syphilis cases presenting at ANC | Number | HMIS |
| HIV prevalence | HIV prevalence among women aged 15-49 | Proportion | DHS |
| Education | % Women aged 15-49 who completed more than secondary education | Proportion | DHS |
| Syphilis testing coverage | Proportion of all women attending ANC that were tested. Total syphilis tests done was given by the sum of positive and negative cases | Proportion | HMIS |
| Electricity | % Households with access to electricity | Proportion | DHS |
| Median age at birth | Median age at first birth in women aged 20-49 | Integer | DHS |
| Number of sex partners | Reported number of sex partners | Integer | DHS |
| % Women employed | % Women employed in the last 12 months | Proportion | DHS |


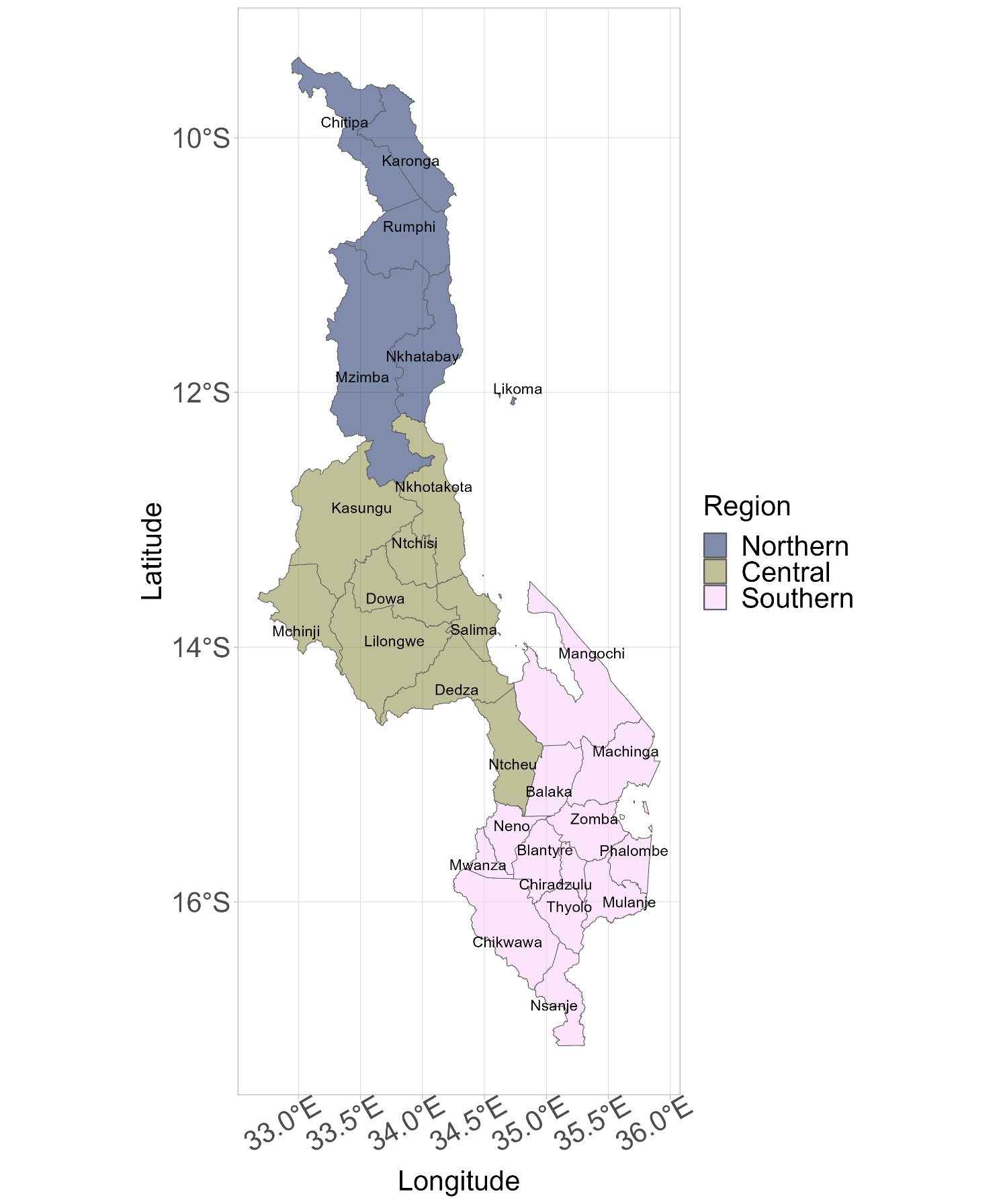


Figure 1: Map of Malawi showing the location of all the 28 districts that contributed the maternal syphilis data
